# Comparative efficacy of electrical stimulation therapies for obstructive sleep apnea: A network meta-analysis of randomized controlled trials

**DOI:** 10.1097/MD.0000000000044103

**Published:** 2025-08-29

**Authors:** Mingfeng Wang, Xiaoming Yin, Yuming Liang, Cunyun Zhang, Xiaofei Zhang, Xiaochun Jiang, Ting Hu

**Affiliations:** a Department of General Practice, Anqing Municipal Hospital, Anqing, Anhui, China; b Anqing Medical College, Anqing, Anhui, China.

**Keywords:** Apnea–Hypopnea Index, electrical stimulation therapy, meta-analysis, obstructive sleep apnea, oxygen saturation, submental electrical stimulation

## Abstract

**Background:**

The objective of this study was to compare the efficacy of hypoglossal nerve stimulation (HNS), submental electrical stimulation (SMES), and transcutaneous electrical nerve stimulation (TENS) on key outcomes in obstructive sleep apnea (OSA).

**Methods:**

A network meta-analysis was conducted using data from 12 randomized controlled trials involving 677 OSA patients. Trials were identified through a systematic search of PubMed, Embase, and Cochrane Library databases up to June 2024. Effect sizes were calculated using standardized mean differences (SMDs) with 95% confidence intervals (CIs) for Apnea–Hypopnea Index (AHI), oxygen saturation, Oxygen Desaturation Index, lowest oxygen saturation during sleep (LSAT), and Epworth Sleepiness Scale. Random-effects models were used to synthesize the data and estimate both direct and indirect effects.

**Results:**

SMES exhibited the greatest reduction in AHI (SMD = –1.14, 95% CI [–1.51, –0.78]) and was most effective for improving oxygen saturation (SMD = 0.80, 95% CI [0.48, 1.12]) and ODI (SMD = –0.92, 95% CI [–1.27, –0.57]). TENS significantly improved LSAT (SMD = 0.68, 95% CI [0.30, 1.06]). HNS produced the largest improvement in Epworth Sleepiness Scale (SMD = –1.03, 95% CI [–1.53, –0.53]).

**Conclusion:**

SMES, TENS, and HNS are effective treatments for OSA. SMES is physiologically superior for reducing AHI and improving oxygenation, owing to its ability to stimulate multiple upper-airway muscles and induce long-term neuromuscular adaptation. TENS improves LSAT by enhancing respiratory muscle performance, and HNS effectively reduces daytime sleepiness. These therapies offer modality-specific benefits that may support personalized OSA management.

## 1. Introduction

Obstructive sleep apnea (OSA) is a prevalent sleep disorder characterized by recurrent episodes of upper airway collapse during sleep, leading to intermittent cessation of breathing (apnea) and reduced airflow (hypopnea).^[1]^ The clinical manifestations of OSA include loud snoring, fragmented sleep, excessive daytime sleepiness, and cognitive impairment.^[2]^ Beyond these symptoms, OSA is associated with a heightened risk for a range of comorbidities, including hypertension, cardiovascular disease, stroke, and metabolic disorders such as diabetes.^[3]^ The global burden of OSA has increased significantly, with millions of individuals affected worldwide, thereby imposing substantial societal and economic costs due to increased healthcare utilization and lost productivity.^[4]^

The management of OSA typically involves nonsurgical interventions such as continuous positive airway pressure (CPAP) therapy and oral appliances.^[5]^ While these treatments are effective for many patients, their limitations (such as low adherence to CPAP therapy and variable effectiveness of oral appliances) have prompted the exploration of alternative treatment options.^[6,7]^ Recently, electrical stimulation therapies have garnered increasing attention as potential treatments for OSA. These therapies utilize electrical currents to stimulate nerves and muscles in the upper airway, with the aim of improving muscle tone and preventing airway collapse during sleep.^[8,9]^ Among the various electrical stimulation modalities, transcutaneous electrical nerve stimulation (TENS), hypoglossal nerve stimulation (HNS), and submental electrical stimulation (SMES) have been investigated for their potential to alleviate OSA symptoms.^[10,11]^

Several meta-analyses have evaluated the efficacy of electrical stimulation therapies in OSA patients, but the results have been mixed. Byun et al conducted a meta-analysis that demonstrated TENS could significantly improve the Apnea–Hypopnea Index (AHI), suggesting a reduction in apneic events. However, this study did not observe significant improvements in other important clinical outcomes, such as daytime sleepiness or overall sleep quality.^[12]^ In contrast, Braun et al focused on HNS and reported significant improvements in the Epworth Sleepiness Scale (ESS) scores, a measure of daytime sleepiness, and overall sleep quality, suggesting that HNS not only reduces apneic events but also improves subjective sleep quality and daytime functioning.^[13]^ These studies highlight the potential benefits of electrical stimulation therapies but also reveal significant variability in outcomes across different interventions.

Despite these promising findings, there remains a lack of consensus regarding the relative efficacy of different electrical stimulation therapies. The variability in results underscores the need for a comprehensive evaluation that systematically compares the various modalities. Moreover, there is a notable absence of studies that have systematically synthesized the available evidence from randomized controlled trials (RCTs) to directly compare the efficacy of TENS, HNS, and SMES in treating OSA. A network meta-analysis (NMA) offers a unique opportunity to compare these therapies head-to-head and provide a more robust evaluation of their relative efficacy.

The aim of this study is to conduct a NMA to compare the efficacy of different electrical stimulation therapies for OSA based on data from RCTs. By synthesizing the available evidence, this study seeks to provide a comprehensive assessment of the effectiveness of TENS, HNS, and SMES in improving key clinical outcomes in OSA patients. The findings from this analysis will help guide clinical decision-making and inform the selection of optimal electrical stimulation therapies for the management of OSA, ultimately improving patient outcomes and quality of life.

## 2. Methods

This systematic review and NMA was conducted in accordance with the Preferred Reporting Items for Systematic Reviews and Meta-Analyses (PRISMA) 2020 guidelines and the PRISMA Extension for NMA (PRISMA-NMA).^[14,15]^ As a meta-analysis of published studies, this research did not require ethical approval or consent.

### 2.1. Data sources and search strategy

A comprehensive literature search was conducted in PubMed, Embase, the Cochrane Central Register of Controlled Trials, Web of Science, and China National Knowledge Infrastructure databases. The search was performed from the inception of each database to December 31, 2024, with no language restrictions. Search terms included “obstructive sleep apnea (OSA),” and various electrical stimulation therapies, such as “transcutaneous electrical nerve stimulation (TENS),” “Hypoglossal Nerve Stimulation (HNS),” and “Submental Electrical Stimulation (SMES).” Detailed search strategies, including keywords, dates, and methods, are provided in Table S1, Supplemental Digital Content, https://links.lww.com/MD/P763. Additionally, we manually screened the reference lists of relevant articles and reviews to identify further studies. Screening of titles, abstracts, and full texts was independently performed by 2 researchers. Discrepancies were resolved by discussion or adjudication by a third author.

### 2.2. Study selection

We included studies that met the following criteria: adult patients (≥18 years) diagnosed with OSA; interventions involving any type of electrical stimulation therapy, such as TENS, PNS, or SNS; controls receiving placebo treatments or non-electrical, nonsurgical interventions, such as CPAP or oral appliance therapy; outcomes including the AHI, Oxygen Desaturation Index (ODI), Epworth Sleepiness Scale (ESS) score, and the Functional Outcomes of Sleep Questionnaire score; designed as RCTs. Studies involving animal models, surgical interventions, case reports, reviews, duplicate publications, or those with incomplete outcome data or flawed statistical methods were excluded. Two independent reviewers assessed the eligibility of studies based on titles, abstracts, and full texts according to the inclusion and exclusion criteria.

### 2.3. Data extraction

Following the literature search, all relevant articles were managed using EndNote X9. Data extraction was performed independently by 2 authors for studies meeting the inclusion criteria. Any disagreements were resolved through consensus or discussion with a third author. Extracted data included publication details (authors, title, year, journal), sample size, demographic characteristics (age, gender), intervention details, and outcome measures. For studies reporting standard errors (SE) for experimental and control groups, standard deviations (SD) were calculated using the formula SD = SE × √n. When SE were not provided, we estimated SDs using guidance from section 7.7.3 of the Cochrane Handbook for Systematic Reviews of Interventions, based on confidence intervals, t-values, interquartile ranges, ranges, or *P*-values. If critical data were missing, we contacted the corresponding authors up to 4 times within 6 weeks to obtain the necessary information.

### 2.4. Risk of bias assessment

The risk of bias in the included studies was independently assessed by 2 authors using the revised Cochrane Risk of Bias Tool (RoB 2).^[16]^ This assessment focused on key domains, including the randomization process, intervention bias, missing outcome data, outcome measurement, and selective reporting. Discrepancies were resolved through discussion or by consulting a third reviewer to ensure a rigorous and complete evaluation of the risk of bias.

### 2.5. Data coding

Electrical stimulation therapies were categorized and coded based on the type of intervention used in each study. The 3 main categories were: TENS, which involves noninvasive electrical stimulation applied through electrodes placed on the skin; HNS, which involves electrical stimulation of the hypoglossal nerve to stimulate the tongue muscles and prevent airway collapse during sleep; and SMES, which targets the submental area to stimulate the muscles of the tongue and upper airway, reducing the risk of airway obstruction in patients with OSA. Each study was reviewed to determine the specific type of electrical stimulation used, and the corresponding code was assigned. Data coding was carried out independently by 2 reviewers, with any discrepancies resolved through discussion.

### 2.6. Data analysis

Data analysis was performed using Stata software (version 17.0, StataCorp LLC, Texas). A NMA was conducted to compare the efficacy of different electrical stimulation therapies in the treatment of OSA. Network plots were generated to visualize the connections between interventions, ensuring the appropriateness of the NMA structure. A random-effects model was employed to account for both within-study and between-study heterogeneity.

As the measurement methods or units of ODI varied across studies, SMD with 95% confidence intervals (CIs) were used for this outcome, while mean differences (MD) were calculated for the remaining outcomes. The I² statistic was used to assess heterogeneity, with thresholds of 25%, 50%, and 75% indicating low, moderate, and high heterogeneity, respectively. Bayesian NMA was performed in Stata using the “network” and “mvmeta” packages. Treatment rankings were based on the surface under the cumulative ranking curve (SUCRA) values, with higher SUCRA values indicating better relative treatment effects.

Publication bias was assessed by generating adjusted funnel plots and conducting Egger test. A *P*-value < .05 was considered indicative of potential bias.^[17]^ Prediction interval plots were also generated to further explore heterogeneity and explain the variability in effect sizes across studies. All statistical tests were 2-tailed, and *P*-values < .05 were considered statistically significant.

## 3. Results

### 3.1. Characteristics of included studies

**Figure 1.**
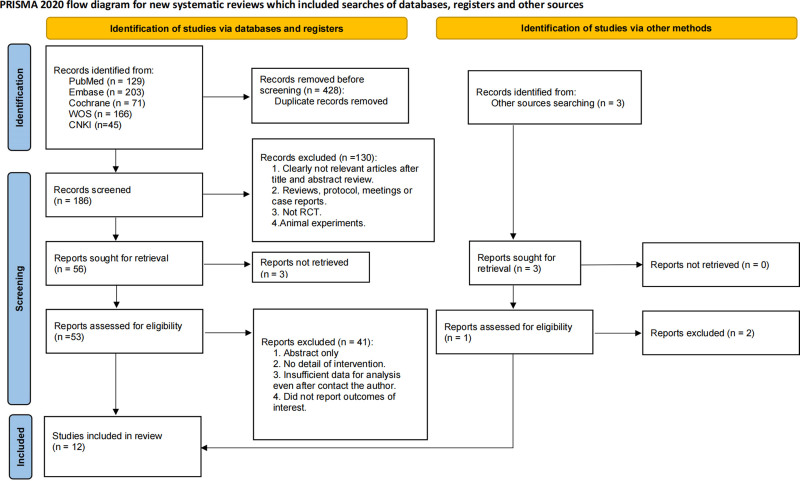
PRISMA flow diagram of the search process for studies. PRISMA = Preferred Reporting Items for Systematic Reviews and Meta-Analyses, RCT = randomized controlled trials.

The initial electronic search identified a total of 614 records. After removing duplicates, 186 records underwent title and abstract screening, and 53 articles were selected for full-text evaluation. Ultimately, 12 studies involving 677 participants met the inclusion criteria and were included in this review (Fig. 1). These studies primarily focused on patients with OSA, with sample sizes ranging from 12 to 138 participants, and a median sample size of 41. Participants’ ages ranged from 27 to 74 years, with a median age of 51.4 years. The publication years of the included studies spanned from 1989 to 2023, with a median publication year of 2005.

Treatment durations varied from 1 day to 15 months. Among the 12 studies, 5 utilized TENS, 4 employed HNS, and 3 used SMES. Compliance with the assigned intervention was generally high across all groups. Reported adherence rates were 97.2% for TENS, 98.7% for HNS, 95.7% for placebo or sham interventions, and 100% for SMES, suggesting good tolerability and feasibility of electrical stimulation therapies in patients with OSA. Detailed study characteristics, including demographic profiles, intervention parameters, and outcome definitions, are summarized in Table S2, Supplemental Digital Content, https://links.lww.com/MD/P763.

### 3.2. The results of NMA

#### 3.2.1. AHI

**Figure 2.**
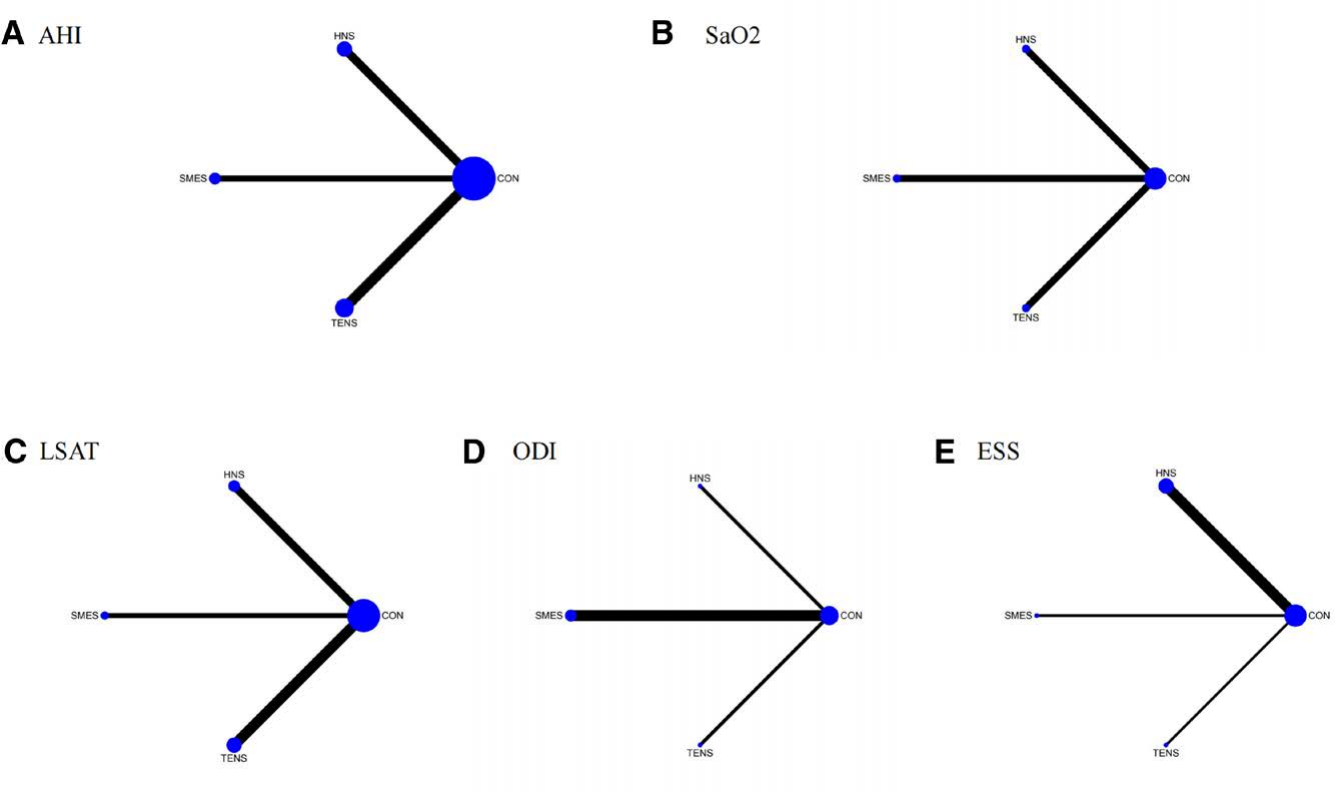
Network of eligible treatment comparisons for cognition. (A) AHI, (B) SaO_2_, (C) LSAT, (D) ODI, and (E) ESS. AHI = Apnea–Hypopnea Index, ESS = Epworth Sleepiness Scale, LSAT = lowest oxygen saturation during sleep, ODI = Oxygen Desaturation Index, SaO_2_ = oxygen saturation.

**Figure 3.**
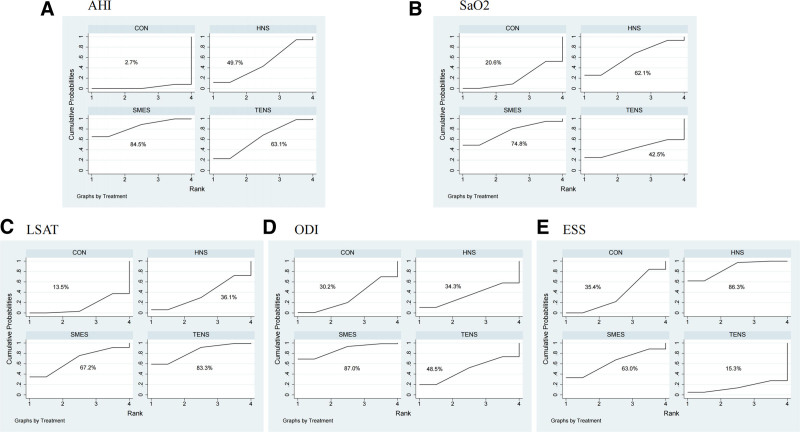
Ranking of exercise strategies based on probability of their effects for cognition. (A) AHI, (B) SaO_2_, (C) LSAT, (D) ODI, and (E) ESS. AHI = Apnea–Hypopnea Index, ESS = Epworth Sleepiness Scale, LSAT = lowest oxygen saturation during sleep, ODI = Oxygen Desaturation Index, SaO_2_ = oxygen saturation.

The NMA of AHI included 12 studies involving 579 OSA patients, evaluating the effects of different electrical stimulation therapies on AHI levels. Figure 2A displays the direct comparisons and sample size distribution across various electrical stimulation interventions. According to the SUCRA ranking (Fig. 3A), the top 3 treatments for improving AHI in OSA patients were SMES (84.5%), TENS (63.1%), and HNS (49.7%), with the control group ranked lowest (2.7%). As shown in Table 1(1), both SMES (MD = −17.51, 95% CI: −30.84 to −4.17) and TENS (MD = −11.97, 95% CI: −22.99 to −0.96) significantly reduced AHI compared to the control group.

**Table 1:** League table of outcomes.

(1) AHI			
SMES			
-5.53 (-22.83, 11.76)	TENS		
-8.24 (-25.74, 9.26)	-2.70 (-18.51, 13.10)	HNS	
-17.51 (-30.84, -4.17)	-11.97 (-22.99, -0.96)	-9.27 (-20.60, 2.06)	CON

(2) SaO_2_			
SMES			
0.10 (-0.49, 0.69)	HNS		
0.24 (-0.71, 1.18)	0.13 (-0.77, 1.04)	TENS	
0.33 (-0.13, 0.79)	0.23 (-0.14, 0.60)	0.09 (-0.73, 0.92)	CON

(3) LSAT			
TENS			
1.66 (-6.81, 10.13)	SMES		
4.52 (-3.03, 12.07)	2.86 (-5.73, 11.45)	HNS	
5.95 (0.70, 11.20)	4.29 (-2.38, 10.97)	1.43 (-3.97, 6.83)	CON

(4) LSAT			
SMES			
-0.84 (-2.96, 1.29)	TENS		
-1.17 (-3.28, 0.94)	-0.33 (-2.78, 2.11)	HNS	
-1.14 (-2.36, 0.07)	-0.31 (-2.05, 1.44)	0.03 (-1.69, 1.75)	CON

(5) ESS			
HNS			
-0.94 (-5.59, 3.70)	SMES		
-2.24 (-3.90, -0.58)	-1.30 (-5.64, 3.04)	CON	
-4.24 (-9.62, 1.13)	-3.30 (-10.01, 3.41)	-2.00 (-7.11, 3.11)	TENS

AHI = Apnea–Hypopnea Index, ESS = Epworth Sleepiness Scale, HNS = hypoglossal nerve stimulation, LSAT = lowest oxygen saturation during sleep, SaO_2_ = oxygen saturation, SMES = submental electrical stimulation, TENS = transcutaneous electrical nerve stimulation.

#### 3.2.2. Oxygen saturation (SaO_2_)

The NMA of SaO_2_ included 6 studies involving 346 OSA patients, assessing the effect of electrical stimulation therapies on SaO_2_ levels. Figure 2B shows the direct comparisons and sample size distribution for the different treatments. According to the SUCRA ranking (Fig. 3B), the top 3 therapies for improving SaO_2_ were SMES (74.8%), HNS (62.1%), and TENS (42.5%), with the control group ranked lowest (20.6%). As indicated in Table 1(2), no statistically significant differences were observed between the different electrical stimulation therapies or compared to the control group.

#### 3.2.3. Lowest saturation (LSAT)

The NMA of LSAT included 9 studies involving 488 OSA patients, evaluating the effects of electrical stimulation therapies on LSAT levels. Figure 2C shows the direct comparisons and sample size distribution. Based on the SUCRA ranking (Fig. 3C), the top 3 treatments for improving LSAT were TENS (83.3%), SMES (67.2%), and HNS (36.1%), with the control group ranked lowest (13.5%). As shown in Table 1(3), TENS significantly improved LSAT compared to the control group (MD = 5.95, 95% CI: 0.70–11.20).

#### 3.2.4. ODI

The NMA of ODI included 5 studies involving 247 OSA patients, assessing the impact of electrical stimulation therapies on ODI. Figure 2D shows the direct comparisons and sample size distribution. According to the SUCRA ranking (Fig. 3D), the top 3 treatments for improving ODI were SMES (87.0%), TENS (48.5%), and HNS (34.3%), with the control group ranked lowest (30.2%). As shown in Table 1(4), no significant differences were found between the different electrical stimulation therapies or compared to the control group.

#### 3.2.5. ESS

The NMA of ESS included 6 studies involving 402 OSA patients, evaluating the effect of electrical stimulation therapies on ESS scores. Figure 2E presents the direct comparisons and sample size distribution. According to the SUCRA ranking (Fig. 3E), the top 2 treatments for reducing ESS scores were HNS (86.3%) and SMES (63.0%), with TENS ranked lowest (15.3%). As shown in Table 1(5), HNS significantly reduced ESS scores compared to the control group (MD = −2.24, 95% CI: −3.90 to −0.58).

### 3.3. Risk of bias and publication bias

The risk of bias assessment for the 12 included studies revealed that 5 studies were considered to have a low overall risk of bias, while 7 studies had some concerns. Regarding the randomization process, 9 studies (75%) were classified as low risk, and 3 studies (25%) had some concerns. For deviations from intended interventions, 7 studies (58.3%) were deemed low risk, and 5 studies (41.7%) had some concerns. In terms of missing outcome data, 9 studies (75%) were considered low risk. All 12 studies were classified as low risk for outcome measurement. Regarding selection of reported results, 10 studies (83.3%) were classified as low risk. Detailed assessments of the risk of bias in each domain are provided in Table S3, Supplemental Digital Content, https://links.lww.com/MD/P763.

Potential publication bias was assessed using funnel plots (Figure S4, Supplemental Digital Content, https://links.lww.com/MD/P764). The scatter plots showed varying degrees of symmetry around the vertical axis, indicating possible publication bias. Specifically, Figures S4.1 and S4.2, Supplemental Digital Content, https://links.lww.com/MD/P764 showed uniform point distribution, while Figures S4.3 and S4.4, Supplemental Digital Content, https://links.lww.com/MD/P764 displayed some degree of bias. Figure S4.5, Supplemental Digital Content, https://links.lww.com/MD/P764 indicated significant bias, with Egger test results < 0.05, suggesting potential publication bias that warrants cautious interpretation. The remaining results of the Egger test were >0.05, indicating no significant publication bias (Figure S4.6, Supplemental Digital Content, https://links.lww.com/MD/P764).

## 4. Discussion

This NMA, comprising 12 RCTs with 677 OSA patients, offers a comprehensive assessment of the efficacy of various electrical stimulation therapies in treating OSA. The main findings are as follows: first, electrical stimulation therapies significantly reduced the AHI, with SMES proving the most effective. Both SMES and TENS significantly lowered AHI compared to the control group, suggesting their potential as effective treatments for reducing sleep-related respiratory disturbances in OSA patients. Second, SMES ranked highest for improving SaO_2_ and ODI, while TENS was most effective in improving the LSAT. Notably, TENS was the only treatment to show a significant improvement in LSAT compared to the control group, indicating its particular efficacy in addressing severe oxygen desaturation episodes during sleep. Third, HNS therapy demonstrated the greatest improvement in the ESS scores, suggesting its potential to reduce daytime sleepiness in OSA patients and improve overall daytime functioning. These findings contribute to the growing body of evidence supporting the use of electrical stimulation therapies in OSA treatment, providing a basis for future research aimed at refining these approaches and optimizing patient outcomes. Mechanistically, these differential effects are attributable to the distinct neuro-anatomical targets, stimulation waveforms and dose–response characteristics of each modality.

Although CPAP is widely recognized as the gold-standard therapy for OSA, direct comparative evidence from the included trials was limited. Specifically, only 2 trials compared electrical stimulation modalities directly with CPAP, which was insufficient to form a robust subgroup analysis focused explicitly on CPAP effectiveness. Therefore, CPAP was categorized within the broader “control” group for statistical consistency within the network structure. Notably, previous large-scale studies have demonstrated CPAP’s ability to reduce AHI by approximately 70% to 90% and significantly normalize nocturnal oxygenation.^[18]^ However, real-world effectiveness is often constrained by suboptimal adherence. In contrast, the electrical stimulation therapies analyzed in this study exhibited consistently high adherence rates (97–100%), suggesting their potential as viable alternatives or adjunctive treatments, particularly in patients who are intolerant of CPAP or have mild-to-moderate OSA. Nonetheless, without sufficient direct comparisons, it remains uncertain whether these electrical stimulation therapies represent a complete alternative or merely complementary treatments. Future well-powered randomized controlled trials directly comparing electrical stimulation therapies against CPAP in clearly stratified OSA populations are essential to clarify their relative positioning in clinical practice.

The AHI serves as a critical metric for assessing the severity of OSA, reflecting the frequency of apneic and hypopneic events during sleep, which are closely linked to the morbidity and mortality rates of OSA patients.^[19]^ This study evaluated the effects of 3 common electrical stimulation therapies (HNS, SMES, and TENS) on AHI. These therapies differ in their mechanisms of action: HNS targets the hypoglossal nerve to prevent airway collapse during sleep,^[20]^ SMES delivers surface currents to the submental triangle to synchronously recruit the genioglossus, geniohyoid, mylohyoid and anterior digastric muscles, and TENS applies low-frequency electrical impulses to modulate pain and potentially improve neural control over respiratory muscles.^[21]^ In this study, both SMES and TENS demonstrated a significant reduction in AHI compared to the control group, with SMES emerging as the most effective treatment. This finding is consistent with previous research showing that electrical stimulation therapies can reduce respiratory disturbances in OSA patients.^[22]^ However, our study adds to the literature by providing a direct comparison of multiple stimulation modalities and highlighting the superior efficacy of SMES.

Physiological rationale for SMES superiority. The improvements in AHI observed with SMES and TENS likely involve modulation of airway‑muscle tone and neural activity, yet their mechanisms differ. SMES recruits a broader constellation of suprahyoid fibers, producing synergistic anterior displacement of the tongue base and hyoid complex, thereby enlarging the velopharyngeal cross‑sectional area and lowering the critical closing pressure (Pcrit) more effectively than HNS, which activates only medial branches of the hypoglossal nerve.^[23]^ Repeated daytime SMES sessions also induce myofibre hypertrophy, increased oxidative capacity and heightened tonic EMG activity that persist during sleep (“training effect”), a phenomenon not reported with HNS or TENS.^[24]^ TENS, on the other hand, may alleviate respiratory‑muscle fatigue and improve diaphragmatic function, but its cutaneous discomfort threshold limits current density, resulting in less robust dilator‑muscle recruitment.^[25]^ Collectively, these anatomical and neurophysiological advantages explain the larger AHI reduction achieved by SMES relative to HNS and TENS in our NMA.

SaO_2_, ODI, and LSAT are critical markers for assessing the severity of OSA and its impact on cardiovascular and respiratory health. SaO_2_ reflects the oxygen level in the blood, ODI measures the frequency of oxygen desaturation events, and LSAT represents the lowest oxygen saturation during sleep, which is key to understanding the risks associated with OSA.^[26]^ In this study, SMES was the most effective treatment for improving both SaO_2_ and ODI, while TENS demonstrated the best effect on LSAT. Notably, TENS was the only therapy to show a statistically significant improvement in LSAT compared to the control group. These results suggest that SMES exerts a more homogeneous stabilizing effect on the upper airway, whereas TENS primarily enhances diaphragmatic and intercostal‑muscle endurance, thereby attenuating the most severe desaturation episodes without substantially altering average oxygenation indices.

The observation that TENS significantly improves LSAT but does not yield a proportionate reduction in AHI suggests a fundamentally different mode of action compared with SMES or HNS. Rather than directly mitigating upper airway collapse, TENS may improve the efficacy of ventilatory effort during partial obstruction by augmenting diaphragmatic recruitment and enhancing thoracoabdominal synchrony. These enhancements may increase tidal volume or minute ventilation during hypopneic events, thereby reducing the severity of desaturation episodes without substantially affecting event frequency.^[27]^ This hypothesis is consistent with prior reports indicating that TENS improves oxygenation and muscle efficiency in patients with neuromuscular or restrictive ventilatory disorders.^[28]^ Thus, the primary effect of TENS may lie in bolstering ventilatory mechanics and gas exchange rather than modifying airway patency, suggesting it may be better suited for OSA phenotypes characterized by oxygen desaturation or respiratory inefficiency rather than collapse-dominant mechanisms.

These results align with previous studies, which have shown that SMES improves SaO_2_ and ODI by enhancing the function of the upper airway muscles, thus preventing airway collapse during sleep.^[29]^ TENS, meanwhile, is believed to improve diaphragmatic and respiratory muscle activity, leading to better oxygen exchange and enhanced LSAT.^[30]^ However, the lack of significant improvement in SaO_2_ and ODI with TENS may reflect its more generalized effects on respiratory muscles, rather than a specific impact on the upper airway. The superior performance of SMES in improving SaO_2_ and ODI can be attributed to its targeted stimulation of the upper airway muscles, particularly the genioglossus, which helps maintain airway patency.^[31,32]^ TENS, while effective for LSAT, likely enhances diaphragmatic and intercostal muscle function, which may improve respiratory efficiency and reduce hypoventilation, thus positively influencing LSAT but not other oxygenation metrics.^[28]^ This study highlights the differential effects of electrical stimulation therapies on various oxygenation parameters in OSA patients. Tailored interventions that consider the specific physiological effects of each therapy may optimize treatment outcomes, and further research into the mechanisms behind these therapies is needed to refine their application.

In addition to assessing the impact of electrical stimulation therapies on oxygenation parameters, this study also evaluated their effects on sleep-related outcomes, particularly the ESS score, a widely used measure of daytime sleepiness in OSA patients. Excessive daytime sleepiness, as reflected by a high ESS score, is a key clinical feature of OSA and is closely linked to impaired cognitive function, poor quality of life, and increased accident risk.^[33]^ Among the therapies analyzed, HNS demonstrated the most significant improvement in ESS scores. This finding underscores the potential of HNS in alleviating excessive daytime sleepiness, a common and debilitating symptom in OSA patients. HNS likely works by stimulating the upper airway muscles during sleep, improving airway patency and reducing the frequency and severity of apneic events. By reducing nighttime sleep disruptions, HNS may help enhance overall sleep quality and, consequently, reduce daytime sleepiness.^[34]^ Unlike other electrical stimulation techniques, HNS specifically targets the upper airway, leading to a more pronounced effect on sleep-related outcomes such as ESS. In contrast, therapies like SMES and TENS, while effective for improving oxygenation parameters, may not have the same targeted impact on sleep fragmentation or daytime wakefulness.^[35]^ This analysis provides valuable insight into the potential role of HNS as an effective intervention for managing daytime sleepiness in OSA patients. Further investigation into the long-term effects of HNS on daytime functioning and its mechanisms of action will be essential to optimize its therapeutic application in clinical practice.

This study has several notable strengths. First, by including only RCTs, the analysis minimizes bias and ensures high-quality evidence. RCTs provide a rigorous framework for assessing treatment efficacy, offering reliable and valid results. Second, the use of NMA allows for a comprehensive comparison of various electrical stimulation therapies within a single model, enabling the estimation of both direct and indirect effects. This approach enhances the robustness of the findings and facilitates more accurate conclusions regarding the relative efficacy of each treatment. Nevertheless, this study has several limitations. First, although all included studies were RCTs, the relatively small number (n = 12) and high variability in patient characteristics (including age, OSA severity, BMI, and comorbidities) as well as protocol parameters such as electrode placement, stimulation intensity, frequency, and session duration, introduced substantial clinical and methodological heterogeneity. While a random-effects model and SUCRA-based rankings were employed to account for between-study variability, such heterogeneity may still have influenced treatment comparability and effect estimates. In addition, most studies did not report stratified outcomes by OSA severity, and baseline AHI data were inconsistently described, precluding formal subgroup analysis. Moreover, inconsistent reporting of stimulation parameters prevented exploration of protocol-dependent effects via subgroup or meta-regression analyses. These limitations highlight the urgent need for future RCTs to adopt standardized stimulation protocols, uniform reporting practices, and stratified analyses to better guide individualized therapy. Second, most included studies assessed outcomes within relatively short follow-up periods (ranging from 1 day to a few months), precluding conclusions about the durability of treatment effects. Although long-term data for HNS are available from studies such as Braun et al and Alrubasy et al,^[13,35]^ demonstrating sustained AHI and ESS improvements up to 5 years, there remains a significant lack of extended follow-up data for both SMES and TENS. This limits our ability to compare their long-term efficacy. Future randomized trials with ≥ 12-month follow-up are urgently needed to confirm whether the benefits of SMES and TENS persist over time. Third, adherence to electrical‑stimulation regimens was rarely reported, yet non‑compliance may attenuate real‑world effectiveness. Future studies should include adherence as a key variable to better understand its impact on treatment success. Fourth, potential publication bias was identified, particularly for the LSAT outcomes, as indicated by funnel plots and Egger test. Such bias might have inflated effect sizes in favor of electrical stimulation therapies, warranting cautious interpretation of these specific results. To mitigate potential bias in future research, we recommend conducting larger, rigorously designed randomized controlled trials with comprehensive reporting of both positive and negative findings. Despite these limitations, this study provides important insights into the efficacy of electrical stimulation therapies for OSA, offering a foundation for future research on optimizing treatment protocols, improving patient adherence, and assessing long-term outcomes.

## 5. Conclusion

This NMA provides robust evidence supporting the efficacy of electrical stimulation therapies in the management of OSA. Among the therapies evaluated, SMES demonstrated the greatest reduction in AHI and improvement in SaO2 and ODI, highlighting its superior ability to enhance upper airway muscle tone and prevent airway collapse. TENS, while less effective in improving AHI, showed significant improvements in LSAT, indicating its potential for addressing severe oxygen desaturation during sleep. HNS therapy was most effective in reducing daytime sleepiness, as reflected by significant improvements in ESS scores. These findings underscore the potential of electrical stimulation therapies, particularly SMES and TENS, as valuable interventions in OSA treatment. Future studies should focus on long-term efficacy, patient adherence, and further mechanistic insights to refine treatment protocols and optimize clinical outcomes.

## Acknowledgments

The authors thank the financial support of the Anqing Municipal Hospital College-level Project Fund (2021aqykj12) and the Project Fund of Southern Anhui Medical College (JXYY202241).

## Author contributions

**Conceptualization:** Ting Hu.

**Data curation:** MingFeng Wang, XiaoMing Yin, Yuming Liang, CunYun Zhang, XiaoFei Zhang, Xiaochun Jiang, Ting Hu.

**Formal analysis:** MingFeng Wang, Ting Hu.

**Funding acquisition:** Ting Hu.

**Project administration:** Ting Hu.

**Resources:** XiaoMing Yin, XiaoFei Zhang, Ting Hu.

**Software:** MingFeng Wang, Yuming Liang.

**Supervision:** MingFeng Wang, XiaoMing Yin, XiaoFei Zhang.

**Validation:** MingFeng Wang, Xiaochun Jiang.

**Writing – original draft:** MingFeng Wang, XiaoMing Yin, XiaoFei Zhang, Ting Hu.

**Writing – review & editing:** Xiaochun Jiang, Ting Hu.

## Supplementary Material



## References

[1] Rundo JV. Obstructive sleep apnea basics. Cleve Clin J Med. 2019;86(9 Suppl 1):2–9. 31509498 10.3949/ccjm.86.s1.02

[2] Borel AL. Sleep apnea and sleep habits: relationships with metabolic syndrome. Nutrients. 2019;11:2628. 31684029 10.3390/nu11112628 PMC6893600

[3] Liu J Yang X Li G Liu P. Pharmacological interventions for the treatment of obstructive sleep apnea syndrome. Front Med (Lausanne). 2024;11:1359461. 38495117 10.3389/fmed.2024.1359461 PMC10943699

[4] McNicholas WT Korkalainen H. Translation of obstructive sleep apnea pathophysiology and phenotypes to personalized treatment: a narrative review. Front Neurol. 2023;14:1239016. 37693751 10.3389/fneur.2023.1239016 PMC10483231

[5] AlRumaih HS Baba NZ AlShehri A AlHelal A Al-Humaidan A. Obstructive sleep apnea management: an overview of the literature. J Prosthodont. 2018;27:260–5. 27598517 10.1111/jopr.12530

[6] Qiao M Xie Y Wolff A Kwon J. Long term adherence to continuous positive Airway pressure in mild obstructive sleep apnea. BMC Pulm Med. 2023;23:320. 37658304 10.1186/s12890-023-02612-3 PMC10472589

[7] Møkleby M Øverland B. Long-term use of CPAP in patients with obstructive sleep apnea: a prospective longitudinal cohort study. Sleep Biol Rhythms. 2022;20:239–46. 38469264 10.1007/s41105-021-00361-6 PMC10899904

[8] Hsieh Y-H Schell AE Yeh E Strohl MP Curado TF Strohl KP. Neurostimulation in the management of obstructive sleep apnea. Curr Sleep Med Rep. 2022;8:168–79.

[9] Pengo M Schwarz EI Steier J. Electrical stimulation in obstructive sleep apnoea: the less invasive the better? Eur Respir J. 2020;55:1902013. 32029643 10.1183/13993003.02013-2019

[10] Johnson MI Jones G Paley CA Wittkopf PG. The clinical efficacy of transcutaneous electrical nerve stimulation (TENS) for acute and chronic pain: a protocol for a meta-analysis of randomised controlled trials (RCTs). BMJ Open. 2019;9:e029999. 10.1136/bmjopen-2019-029999 PMC6830670 31662366

[11] Soták M Roubík K Henlín T Tyll T. Phrenic nerve stimulation prevents diaphragm atrophy in patients with respiratory failure on mechanical ventilation. BMC Pulm Med. 2021;21:314. 34625059 10.1186/s12890-021-01677-2 PMC8500254

[12] Byun YJ Yan F Nguyen SA Lentsch EJ. Transcutaneous electrical stimulation therapy in obstructive sleep apnea: a systematic review and meta-analysis. Otolaryngol Head Neck Surg. 2020;163:645–53. 32366179 10.1177/0194599820917631

[13] Braun M Stoerzel M Wollny M Schoebel C Ulrich Sommer J Heiser C. Patient-reported outcomes with hypoglossal nerve stimulation for treatment of obstructive sleep apnea: a systematic review and meta-analysis. Eur Arch Otorhinolaryngol. 2023;280:4627–39. 37354340 10.1007/s00405-023-08062-1 PMC10477259

[14] Hutton B Salanti G Caldwell DM. The PRISMA extension statement for reporting of systematic reviews incorporating network meta-analyses of health care interventions: checklist and explanations. Ann Intern Med. 2015;162:777–84. 26030634 10.7326/M14-2385

[15] Page MJ McKenzie JE Bossuyt PM. The PRISMA 2020 statement: an updated guideline for reporting systematic reviews. BMJ. 2021;372:n71. 33782057 10.1136/bmj.n71 PMC8005924

[16] Sterne JAC Savović J Page MJ. RoB 2: a revised tool for assessing risk of bias in randomised trials. BMJ. 2019;366:l4898. 31462531 10.1136/bmj.l4898

[17] Egger M Davey Smith G Schneider M Minder C. Bias in meta-analysis detected by a simple, graphical test. BMJ. 1997;315:629–34. 9310563 10.1136/bmj.315.7109.629 PMC2127453

[18] Van Daele M Smolders Y Van Loo D. Personalized treatment for obstructive sleep apnea: beyond CPAP. Life (Basel). 2024;14:1007. 39202749 10.3390/life14081007 PMC11355307

[19] Tang H Lv F Zhang P Liu J Mao J. The impact of obstructive sleep apnea on nonalcoholic fatty liver disease. Front Endocrinol. 2023;14:1254459. 10.3389/fendo.2023.1254459 PMC10577417 37850091

[20] Hardin L. Hypoglossal nerve stimulation for adults with obstructive sleep apnea. JAAPA. 2023;36:24–9. 10.1097/01.JAA.0000991392.37494.b6 37989167

[21] Zulbaran-Rojas A Bara RO Lee M. Transcutaneous electrical nerve stimulation for fibromyalgia-like syndrome in patients with Long-COVID: a pilot randomized clinical trial. Sci Rep. 2024;14:27224. 39516528 10.1038/s41598-024-78651-5 PMC11549448

[22] Moffa A Giorgi L Carnuccio L. New non-invasive electrical stimulation devices for treatment of snoring and obstructive sleep apnoea: a systematic review. Sleep Breath. 2023;27:103–8. 35460429 10.1007/s11325-022-02615-0

[23] Thuler E Rabelo FAW Yui M Tominaga Q Dos Santos V Jr. Arap SS. Correlation between the transverse dimension of the maxilla, upper airway obstructive site, and OSA severity. J Clin Sleep Med. 2021;17:1465–73. 33688826 10.5664/jcsm.9226 PMC8314622

[24] Caloway CL Diercks GR Keamy D. Update on hypoglossal nerve stimulation in children with down syndrome and obstructive sleep apnea. Laryngoscope. 2020;130:E263–7. 31219619 10.1002/lary.28138

[25] Bjerså K Jildenstaal P Jakobsson J Egardt M Fagevik Olsén M. Adjunct high frequency Transcutaneous Electric Stimulation (TENS) for postoperative pain management during weaning from epidural analgesia following colon surgery: results from a controlled pilot study. Pain Manag Nurs. 2015;16:944–50. 26541070 10.1016/j.pmn.2015.08.006

[26] Wray CM Thaler ER. Hypoglossal nerve stimulation for obstructive sleep apnea: a review of the literature. World J Otorhinolaryngol Head Neck Surg. 2016;2:230–3. 29204571 10.1016/j.wjorl.2016.11.005 PMC5698546

[27] Hsin YF Chen SH Yu TJ Huang CC Chen YH. Effects of transcutaneous electrical diaphragmatic stimulation on respiratory function in patients with prolonged mechanical ventilation. Ann Thorac Med. 2022;17:14–20. 35198044 10.4103/atm.atm_158_21 PMC8809123

[28] Medrinal C Machefert M Lamia B. Transcutaneous electrical diaphragmatic stimulation in mechanically ventilated patients: a randomised study. Crit Care. 2023;27:338. 37649092 10.1186/s13054-023-04597-1 PMC10469422

[29] Baptista PM Martínez Ruiz de Apodaca P Carrasco M. Daytime neuromuscular electrical therapy of tongue muscles in improving snoring in individuals with primary snoring and mild obstructive sleep apnea. J Clin Med. 2021;10:1883. 33925376 10.3390/jcm10091883 PMC8123870

[30] Jamison RN Edwards RR Curran S. Effects of wearable transcutaneous electrical nerve stimulation on fibromyalgia: a randomized controlled trial. J Pain Res. 2021;14:2265–82. 34335055 10.2147/JPR.S316371 PMC8318714

[31] Vranish JR Bailey EF. A comprehensive assessment of genioglossus electromyographic activity in healthy adults. J Neurophysiol. 2015;113:2692–9. 25695653 10.1152/jn.00975.2014 PMC4416595

[32] Zhou N Ho JTF Spijker R. Maxillomandibular advancement and upper airway stimulation for treatment of obstructive sleep apnea: a systematic review. J Clin Med. 2022;11:6782. 36431259 10.3390/jcm11226782 PMC9697253

[33] Chen L Pivetta B Nagappa M. Validation of the STOP-Bang questionnaire for screening of obstructive sleep apnea in the general population and commercial drivers: a systematic review and meta-analysis. Sleep Breath. 2021;25:1741–51. 33507478 10.1007/s11325-021-02299-y PMC8590671

[34] Mashaqi S Patel SI Combs D. The hypoglossal nerve stimulation as a novel therapy for treating obstructive sleep apnea – a literature review. Int J Environ Res Public Health. 2021;18:1642. 33572156 10.3390/ijerph18041642 PMC7914469

[35] Alrubasy WA Abuawwad MT Taha MJJ. Hypoglossal nerve stimulation for obstructive sleep apnea in adults: an updated systematic review and meta-analysis. Respir Med. 2024;234:107826. 39401661 10.1016/j.rmed.2024.107826

