# Supplementary material for: Comparative efficacy of electrical stimulation therapies for obstructive sleep apnea: A network meta-analysis of randomized controlled trials
Source: Medicine (Baltimore). 2025 Aug 29;104(35):e44103. doi: 10.1097/MD.0000000000044103 (PMC12401310; doi:10.1097/MD.0000000000044103)
Supplement: Supplementary file 1 [file medi-104-e44103-s001.docx]

# Supplementary table 1: Search Strategy

***Search Strategy:***

| #13 | Search: (((((((((((((((Sleep Apnea, Obstructive[MeSH Terms]) OR ("Obstructive Sleep Apnoea"[Title/Abstract])) OR (OSA[Title/Abstract])) OR ("Sleep Apnea Syndromes"[Title/Abstract])) OR (Sleep Apnea Syndromes[MeSH Terms])) OR ("Sleep Apnea Syndromes"[Title/Abstract])) OR ("Sleep Apnea"[Title/Abstract])) OR ("Apnoea Syndrome"[Title/Abstract])) OR ("Apnea Syndrome"[Title/Abstract])) OR ("Sleep-disordered Breathing"[Title/Abstract])) OR ("Upper Airway Resistance Syndrome"[Title/Abstract])) OR ("Hypopnea Syndrome"[Title/Abstract])) OR ("Sleep-related Breathing Disorder"[Title/Abstract])) AND (((((((((((((((((((Electric Stimulation Therapy[MeSH Terms]) OR ("Electrical Stimulation"[Title/Abstract])) OR (Transcranial Magnetic Stimulation[MeSH Terms])) OR (TMS[Title/Abstract])) OR ("Repetitive Transcranial Magnetic Stimulation"[Title/Abstract])) OR (rTMS[Title/Abstract])) OR ("Deep Transcranial Magnetic Stimulation"[Title/Abstract])) OR (dTMS[Title/Abstract])) OR ("Theta Burst Stimulation"[Title/Abstract])) OR ("Non-invasive Brain Stimulation"[Title/Abstract])) OR ("Magnetic Brain Stimulation"[Title/Abstract])) OR (Transcutaneous Electrical Nerve Stimulation[MeSH Terms])) OR (TENS[Title/Abstract])) OR (Neuromodulation[Title/Abstract])) OR (Electrotherapy[Title/Abstract])) OR ("Electrical Nerve Stimulation"[Title/Abstract])) OR ("Transdermal Electrical Nerve Stimulation"[Title/Abstract])) OR ("Non-invasive Electrical Stimulation"[Title/Abstract])) OR ("Electrical Nerve Stimulation "[Title/Abstract]))) AND (((((((((Treatment Outcome[MeSH Terms]) OR ("Therapeutic Effect"[Title/Abstract])) OR (Efficacy[Title/Abstract])) OR ("Clinical Efficacy"[Title/Abstract])) OR ("Therapeutic Benefit"[Title/Abstract])) OR (Improvement[Title/Abstract])) OR ("Sleep Quality Improvement"[Title/Abstract])) OR ("Reduction in Symptoms"[Title/Abstract])) OR (Quality of Life[MeSH Terms]))) AND ((randomized controlled trial[pt] OR controlled clinical trial[pt] OR randomized[tiab] OR placebo[tiab] OR drug therapy[sh] OR randomly[tiab] OR trial[tiab] OR groups[tiab]) NOT (animals[mh] NOT humans[mh])) |
| --- | --- |
| #12 | Search: (randomized controlled trial[pt] OR controlled clinical trial[pt] OR randomized[tiab] OR placebo[tiab] OR drug therapy[sh] OR randomly[tiab] OR trial[tiab] OR groups[tiab]) NOT (animals[mh] NOT humans[mh]) |
| #11 | Search: ((((((((Treatment Outcome[MeSH Terms]) OR ("Therapeutic Effect"[Title/Abstract])) OR (Efficacy[Title/Abstract])) OR ("Clinical Efficacy"[Title/Abstract])) OR ("Therapeutic Benefit"[Title/Abstract])) OR (Improvement[Title/Abstract])) OR ("Sleep Quality Improvement"[Title/Abstract])) OR ("Reduction in Symptoms"[Title/Abstract])) OR (Quality of Life[MeSH Terms]) |
| #10 | Search: (((((("Therapeutic Effect"[Title/Abstract])) OR (Efficacy[Title/Abstract])) OR ("Clinical Efficacy"[Title/Abstract])) OR ("Therapeutic Benefit"[Title/Abstract])) OR (Improvement[Title/Abstract])) OR ("Sleep Quality Improvement"[Title/Abstract])) OR ("Reduction in Symptoms"[Title/Abstract]) |
| #9 | Search: (Quality of Life[MeSH Terms]) |
| #8 | Search: (Treatment Outcome[MeSH Terms]) |
| #7 | Search: ((((((((((((((((((Electric Stimulation Therapy[MeSH Terms]) OR ("Electrical Stimulation"[Title/Abstract])) OR (Transcranial Magnetic Stimulation[MeSH Terms])) OR (TMS[Title/Abstract])) OR ("Repetitive Transcranial Magnetic Stimulation"[Title/Abstract])) OR (rTMS[Title/Abstract])) OR ("Deep Transcranial Magnetic Stimulation"[Title/Abstract])) OR (dTMS[Title/Abstract])) OR ("Theta Burst Stimulation"[Title/Abstract])) OR ("Non-invasive Brain Stimulation"[Title/Abstract])) OR ("Magnetic Brain Stimulation"[Title/Abstract])) OR (Transcutaneous Electrical Nerve Stimulation[MeSH Terms])) OR (TENS[Title/Abstract])) OR (Neuromodulation[Title/Abstract])) OR (Electrotherapy[Title/Abstract])) OR ("Electrical Nerve Stimulation"[Title/Abstract])) OR ("Transdermal Electrical Nerve Stimulation"[Title/Abstract])) OR ("Non-invasive Electrical Stimulation"[Title/Abstract])) OR ("Electrical Nerve Stimulation "[Title/Abstract]) |
| #6 | Search: (Transcutaneous Electrical Nerve Stimulation[MeSH Terms]) |
| #5 | Search: (Transcranial Magnetic Stimulation[MeSH Terms]) |
| #4 | Search: (Electric Stimulation Therapy[MeSH Terms]) |
| #3 | Search: ((((((((((((Sleep Apnea, Obstructive[MeSH Terms]) OR ("Obstructive Sleep Apnoea"[Title/Abstract])) OR (OSA[Title/Abstract])) OR ("Sleep Apnea Syndromes"[Title/Abstract])) OR (Sleep Apnea Syndromes[MeSH Terms])) OR ("Sleep Apnea Syndromes"[Title/Abstract])) OR ("Sleep Apnea"[Title/Abstract])) OR ("Apnoea Syndrome"[Title/Abstract])) OR ("Apnea Syndrome"[Title/Abstract])) OR ("Sleep-disordered Breathing"[Title/Abstract])) OR ("Upper Airway Resistance Syndrome"[Title/Abstract])) OR ("Hypopnea Syndrome"[Title/Abstract])) OR ("Sleep-related Breathing Disorder"[Title/Abstract]) |
| #2 | Search: ((((((((((("Obstructive Sleep Apnoea"[Title/Abstract])) OR (OSA[Title/Abstract])) OR ("Sleep Apnea Syndromes"[Title/Abstract])) OR (Sleep Apnea Syndromes[MeSH Terms])) OR ("Sleep Apnea Syndromes"[Title/Abstract])) OR ("Sleep Apnea"[Title/Abstract])) OR ("Apnoea Syndrome"[Title/Abstract])) OR ("Apnea Syndrome"[Title/Abstract])) OR ("Sleep-disordered Breathing"[Title/Abstract])) OR ("Upper Airway Resistance Syndrome"[Title/Abstract])) OR ("Hypopnea Syndrome"[Title/Abstract])) OR ("Sleep-related Breathing Disorder"[Title/Abstract]) |
| #1 | Search: (Sleep Apnea, Obstructive[MeSH Terms]) |

#

# Supplementary table 2: Characteristics of studies and subjects included in the review

| **Study** | **Subjects  (intervention/ control)** | **Sex (male/female) (intervention/ control)** | **Mean age  (intervention/ control)** | **BMI (intervention/ control)** | **Intervention detail** | | **Frequency** | **Follow-up** | **Outcome** |
| --- | --- | --- | --- | --- | --- | --- | --- | --- | --- |
|  |  |  |  |  | **Intervention group** | **Control group** |  |  |  |
| Chwiesko-Minarowska et al. (2016) | 38 (19/19) | 13/6 vs. 14/5 | 55±15 vs. 54±18 | 29.8±5.1 kg/m² vs. 33.1±7.1 kg/m² | TENS | CPAP | Once/day | 4 weeks | AHI, ESS, PSQI |
| Randerath et al. (2004) | 57 (33/34) | 19/14 vs. 25/9 | 50.8±12.1 vs. 53.3±11.3 | 29.1±4.4 kg/m² vs. 28.9±4.9 kg/m² | HNS | Placebo | 2 times/day | 8 weeks | AHI, ESS, LSAT |
| Hida et al. (1994) | 13 (8/5) | 8/0 vs. 5/0 | 51.4±3.2 vs. 53.8±7.3 | 28.1±1.3 kg/m² vs. 26.8±2.8 kg/m² | SMES | Placebo | Once/day | 5 days | AHI, SaO2, ODI |
| Miki et al. (1989) | 12 (6/6) | 5/1 vs. 5/1 | NA | NA | SMES | Placebo | Once/day | 3 days | AHI, LSAT, ODI |
| Pengo et al. (2016) | 72 (32/32) | 30/6 vs. 30/6 | 50.8±11.2 | 29.6±5.9 kg/m² | TENS | Placebo | Once/day | 1 day | AHI, LSAT, SaO2, ODI |
| Steier et al. (2016) | 22 (11/11) | 8/3 vs. 8/3 | 50.7±15.9 | 42.0±9.7 kg/m² | TENS | Placebo | Once/day | 1 day | AHI, LSAT, SaO2 |
| Verse et al. (2003) | 30 (15/15) | 14/1 vs. 14/1 | 59.6±10.7 | 29.3±3.63 kg/m² | SMES | Placebo | Once/day | 4-5 weeks | AHI, ESS, LSAT, SaO2, ODI |
| Heiser et al. (2003) | 89 (45/44) | 37/8 vs. 35/9 | 58.3±9.4 vs. 56.6±10.4 | 28.6±3.7 kg/m² vs. 29.5±3.9 | HNS | Placebo | Once/day | 2 weeks | AHI, ESS, FOSQ, LSAT, SaO2 |
| Pu et al. (1998) | 32 (16/16) | 14/2 vs. 14/2 | 57±12 | 28±3 kg/m² | TENS | Placebo | NA | NA | AHI, LSAT |
| Wang et al. (2021) | 120 (60/60) | 44/16 vs. 42/18 | 50.2±10.8 vs. 49.9±11.2 | 25.7±2.8 kg/m² vs. 25.3±3.0 kg/m² | HNS | CPAP | Once/day | 3 months | AHI, LSAT, SaO2, ODI |
| Hu et al. (2006) | 44 (22/22) | 21/1 vs. 21/1 | 27-74 | 18.62 kg/m²-30.26 kg/m² | TENS | Placebo | Once/day | NA | AHI, LSAT, SaO2 |
| Schwartz et al. (2023) | 138 (92/46) | 75/17 vs. 44/2 | 55.8±8.3 vs. 55.1±10.5 | 29.9±3.0 kg/m² vs. 29.6±3.1 kg/m² | HNS | Placebo | Once/day | 12-15 months | ESS, FOSQ |

NA None available, BMI body mass index, TENS Transcutaneous Electrical Nerve Stimulation, CPAP Continuous Positive Airway Pressure, HNS Hypoglossal Nerve Stimulation, SMES Submental Electrical Stimulation, AHI Apnea-Hypopnea Index, ESS Epworth Sleepiness Scale, PSQI Pittsburgh Sleep Quality Index, SaO2 Oxygen Saturation, LSAT Lowest Oxygen Saturation During Sleep, FOSQ Functional Outcomes of Sleep Questionnaire, ODI Oxygen Desaturation Index.

**List of included studies:**

1. Chwiesko-Minarowska S, Minarowski Ł, Szewczak WA, et al. Efficacy of daytime transcutaneous electrical stimulation of the genioglossus muscle in patients with obstructive sleep apnea syndrome: short report. Head & Neck. 2016 Apr;38(4):653-7. doi: 10.1007/s00405-016-4047-9.
2. Randerath WJ, Galetke W, Domanski U, et al. Tongue-muscle training by intraoral electrical neurostimulation in patients with obstructive sleep apnea. Sleep. 2004 Apr;27(2):254-9. doi: 10.1093/sleep/27.2.254.
3. Hida W, Okabe S, Miki H, et al. Effects of submental stimulation for several consecutive nights in patients with obstructive sleep apnea. Thorax. 1994 May;49(5):446-52. doi: 10.1136/thx.49.5.446.
4. Miki H, Hida W, Chonan T, et al. Effects of Submental Electrical Stimulation during Sleep on Upper Airway Patency in Patients with Obstructive Sleep Apnea. Am Rev Respir Dis. 1989 Oct;140(4):1285-9.
5. Pengo MF, Xiao S, Ratneswaran C, et al. Randomised sham-controlled trial of transcutaneous electrical stimulation in obstructive sleep apnoea. Thorax. 2016 Oct;71(10):923-31. doi: 10.1136/thoraxjnl-2016-208691.
6. Steier J, Seymour J, Rafferty GF, et al. Continuous Transcutaneous Submental Electrical Stimulation in Obstructive Sleep Apnea: A Feasibility Study. Chest. 2011 Oct;140(4):998-1007. doi: 10.1378/chest.10-2614.
7. Verse T, Schwalb JS, Hörmann K, et al. Transkutane, submentale Elektrostimulationstherapie bei obstruktiver Schlafapnoe. HNO. 2003 Dec;51(12):966-70. doi: 10.1007/s00106-003-0842-x.
8. Heiser C, Steffen A, Hofauer B, et al. Effect of Upper Airway Stimulation in Patients with Obstructive Sleep Apnea (EFFECT): A Randomized Controlled Crossover Trial. J Clin Med. 2021 Oct;10(10):2880. doi: 10.3390/jcm10132880.
9. Piao Shang, He Yinfeng, Yuan Fuxiang, et al. "Effects of Transcutaneous Electrical Stimulation on Obstructive Sleep Apnea Syndrome." Chinese Journal of Tuberculosis and Respiratory Diseases. 1998;21(8):492-3.
10. Wang Haibo, Jiang Zongliang, Sun Beibei, et al. "Impact of Transcutaneous Submental Muscle Electrical Stimulation Therapy on the Efficacy and Quality of Life in Patients with Mild to Moderate Obstructive Sleep Apnea-Hypopnea Syndrome (OSAHS)." Medical Research. 2021;50(1):98-101.
11. Hu Lianggang, Xu Xiaomei, Gong Yongsheng, et al. "Experimental Study and Clinical Efficacy of Non-invasive Percutaneous Electrical Stimulator for Obstructive Sleep Apnea Syndrome (OSAS) Treatment." Journal of Biomedical Engineering. 2006;23(4):852-5.
12. Schwartz AR, Jacobowitz O, Eisele DW, et al. Targeted Hypoglossal Nerve Stimulation for Patients With Obstructive Sleep Apnea: A Randomized Clinical Trial. JAMA Otolaryngol Head Neck Surg. 2023;149(6):512-20. doi: 10.1001/jamaoto.2023.0161.

# Supplementary table 3: Risk of Bias

## Table 3.1 The risk of bias assessment for the individual included studies

| **Study** | Randomization process | Deviations from intended interventions | Missing outcome data | Measurement of the outcome | Selection of the reported result | Overall Bias |
| --- | --- | --- | --- | --- | --- | --- |
| Chwiesko-Minarowska et al. (2016) | Some concerns | Low | Some concerns | Low | Some concerns | Some concerns |
| Randerath et al. (2004) | Low | Some concerns | Low | Low | Low | Some concerns |
| Hida et al. (1994) | Low | Low | Low | Low | Low | Low |
| Miki et al. (1989) | Some concerns | Low | Low | Low | Low | Some concerns |
| Pengo et al. (2016) | Low | Some concerns | Low | Low | Some concerns | Some concerns |
| Steier et al. (2016) | Low | Low | Low | Low | Low | Low |
| Verse et al. (2003) | Low | Low | Low | Low | Low | Low |
| Heiser et al. (2003) | Low | Some concerns | Some concerns | Low | Low | Some concerns |
| Pu et al. (1998) | Low | Low | Low | Low | Low | Low |
| Wang et al. (2021) | Low | Some concerns | Some concerns | Low | Low | Some concerns |
| Hu et al. (2006) | Some concerns | Some concerns | Low | Low | Low | Some concerns |
| Schwartz et al. (2023) | Low | Low | Low | Low | Low | Low |

# 
