# Supplementary material for: Comparative efficacy of electrical stimulation therapies for obstructive sleep apnea: A network meta-analysis of randomized controlled trials
Source: Medicine (Baltimore). 2025 Aug 29;104(35):e44103. doi: 10.1097/MD.0000000000044103 (PMC12401310; doi:10.1097/MD.0000000000044103)
Supplement: Supplementary file 2 [file medi-104-e44103-s002.docx]

# Supplementary figure 4: Publication bias


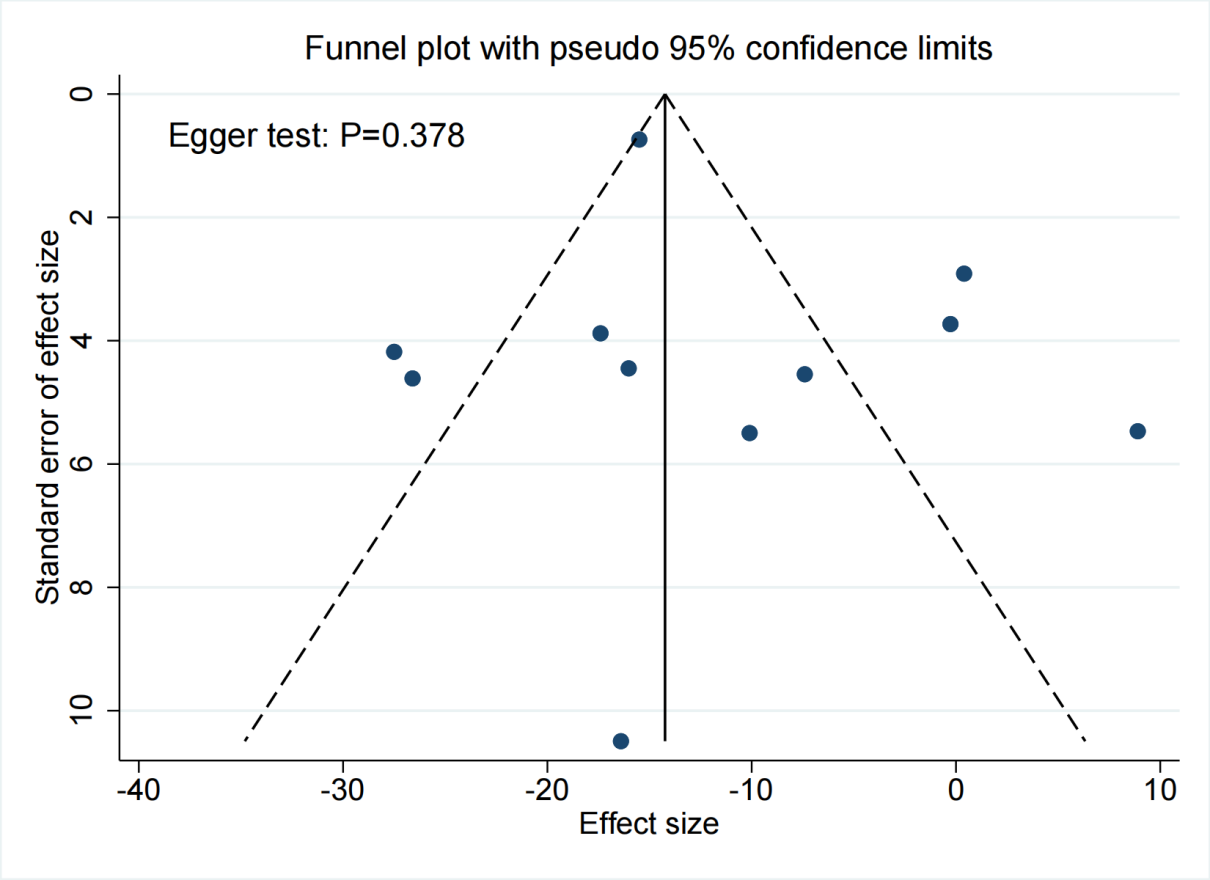


Supplementary figure 4.1 The funnel plot of AHI. The result of Egger test showed the p=0.378.

# Supplementary figure 4: Publication bias


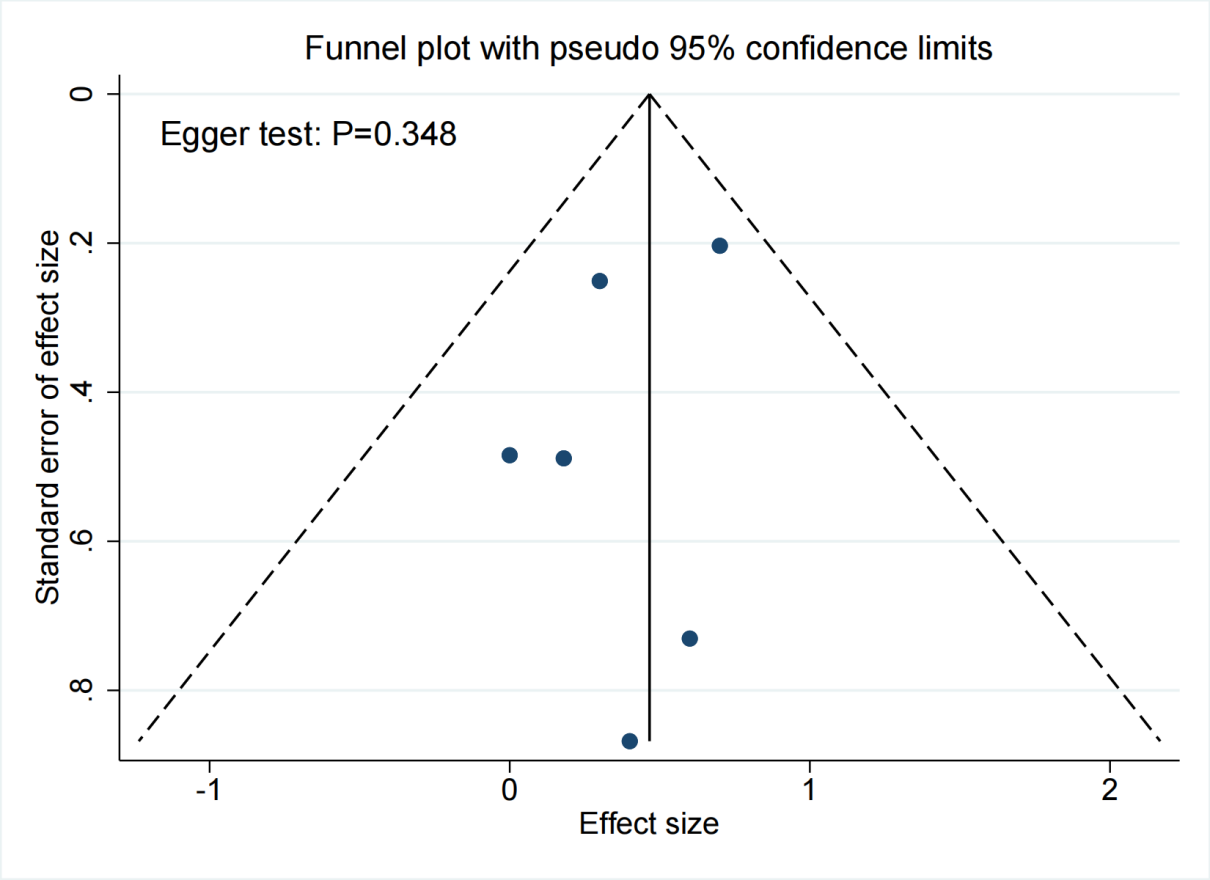


Supplementary figure 4.2 The funnel plot of SaO2. The result of Egger test showed the p=0.348.

# Supplementary figure 4: Publication bias


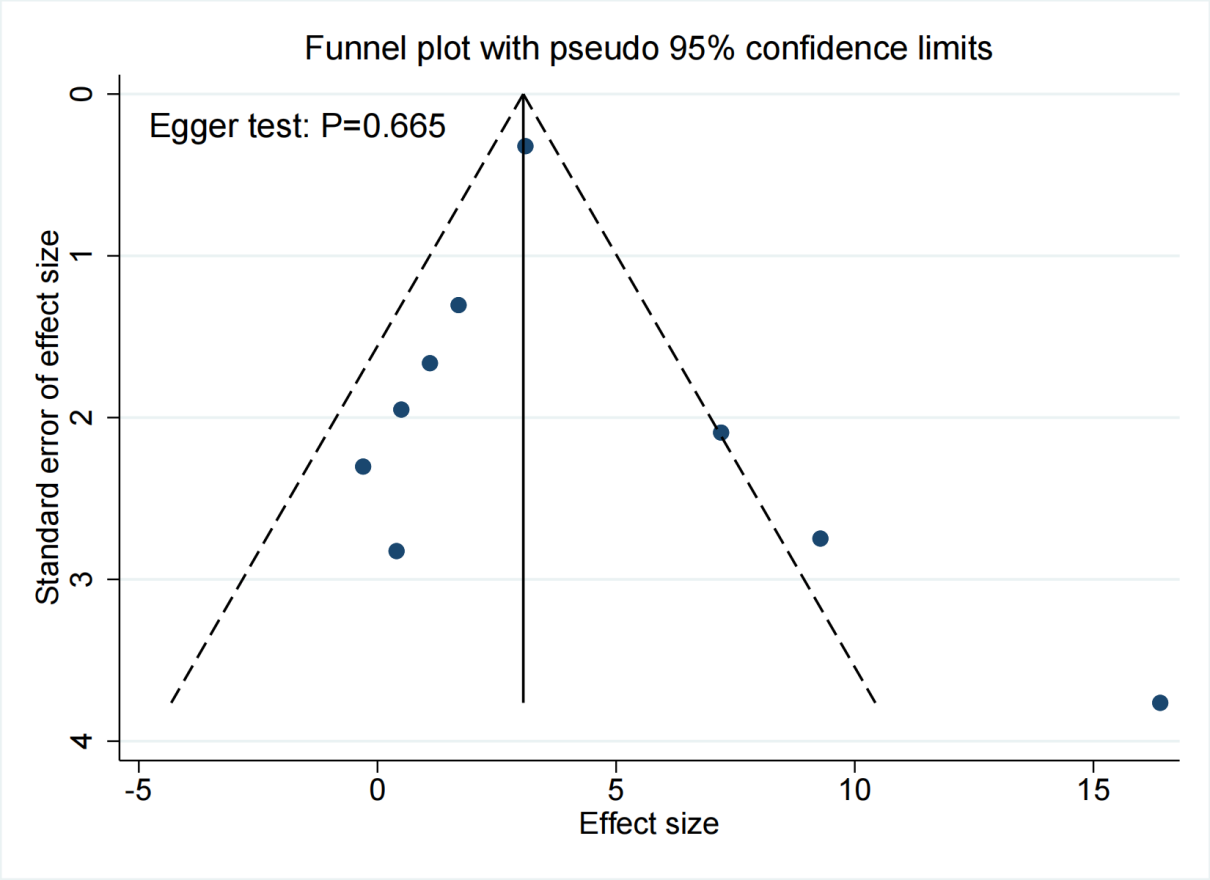


Supplementary figure 4.3 The funnel plot of LSAT. The result of Egger test showed the p=0.665.

# Supplementary figure 4: Publication bias


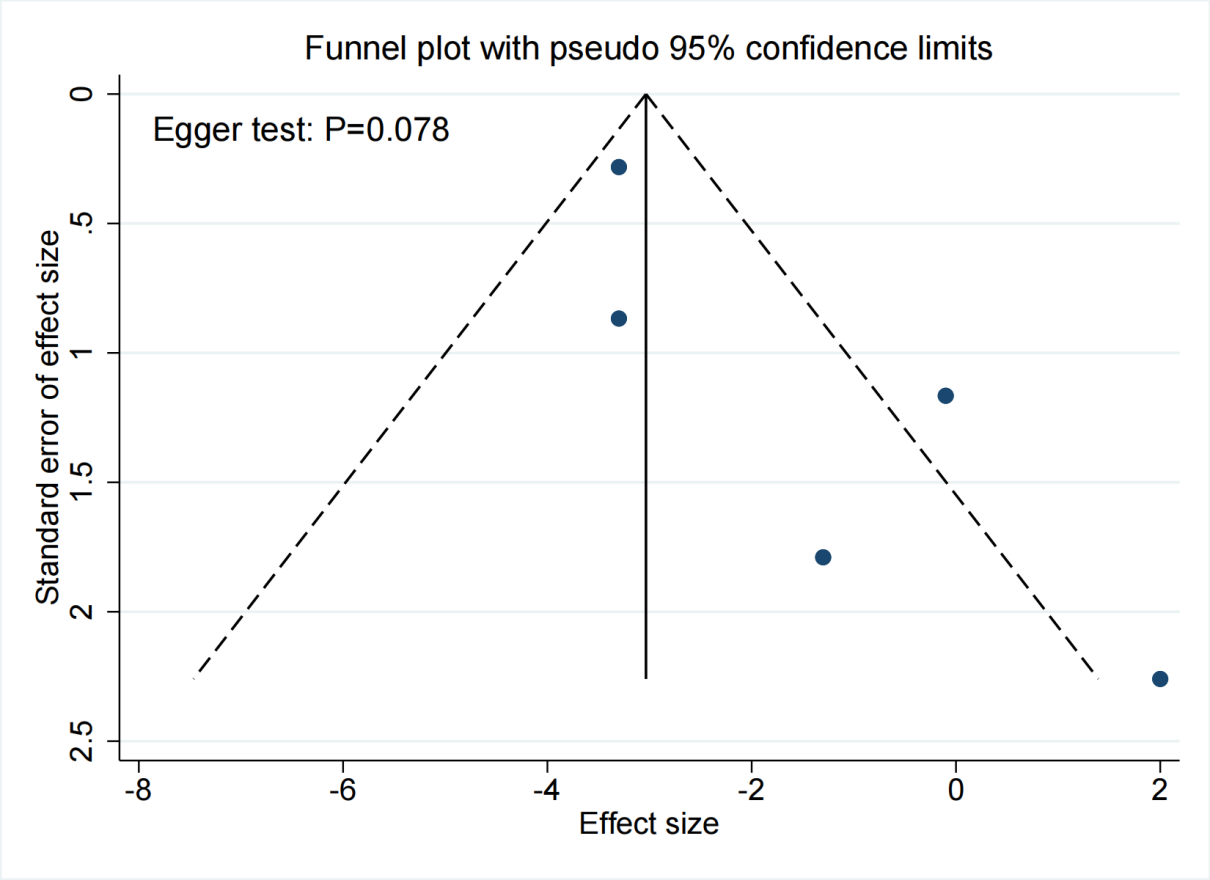


Supplementary figure 4.4 The funnel plot of ESS. The result of Egger test showed the p=0.07.

# Supplementary figure 4: Publication bias


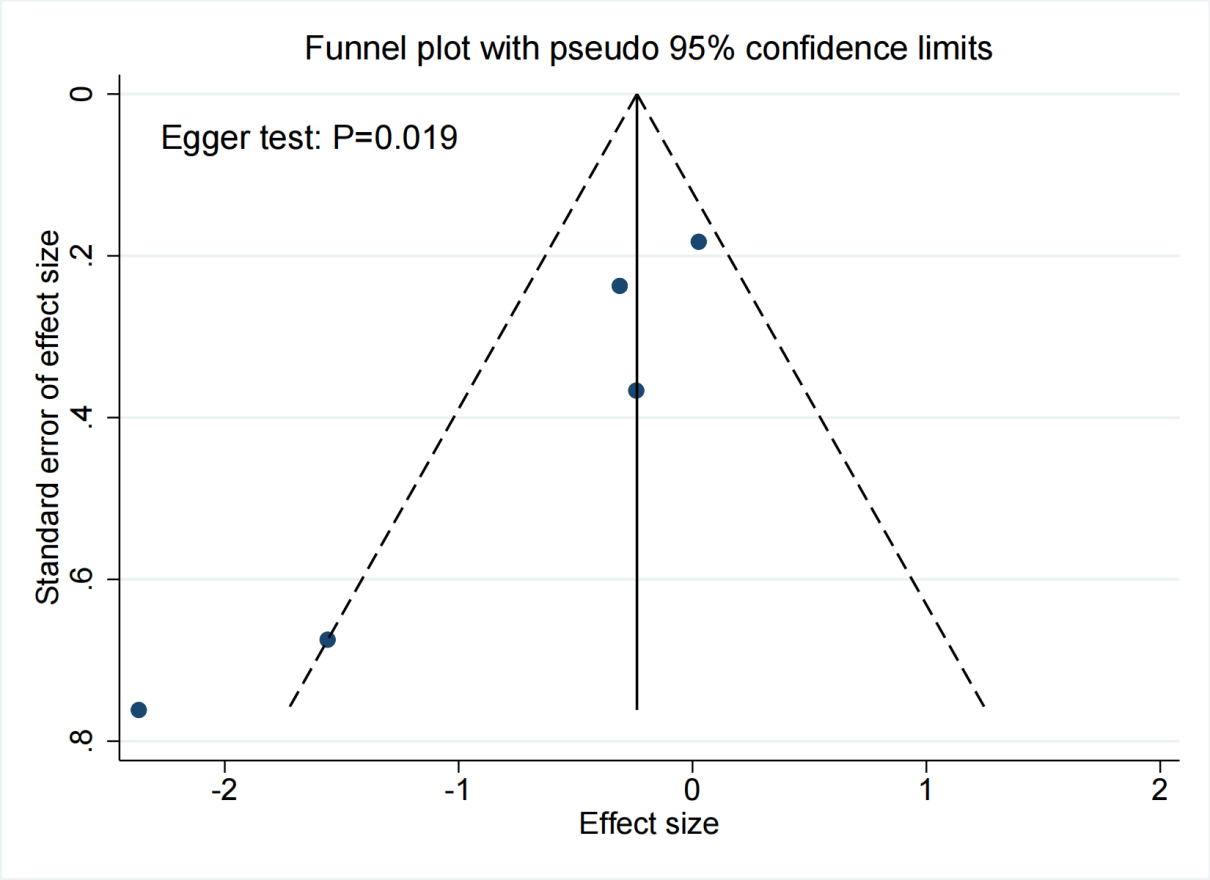


Supplementary figure 4.5 The funnel plot of ODI. The result of Egger test showed the p=0.019.

# Supplementary figure 4: Publication bias


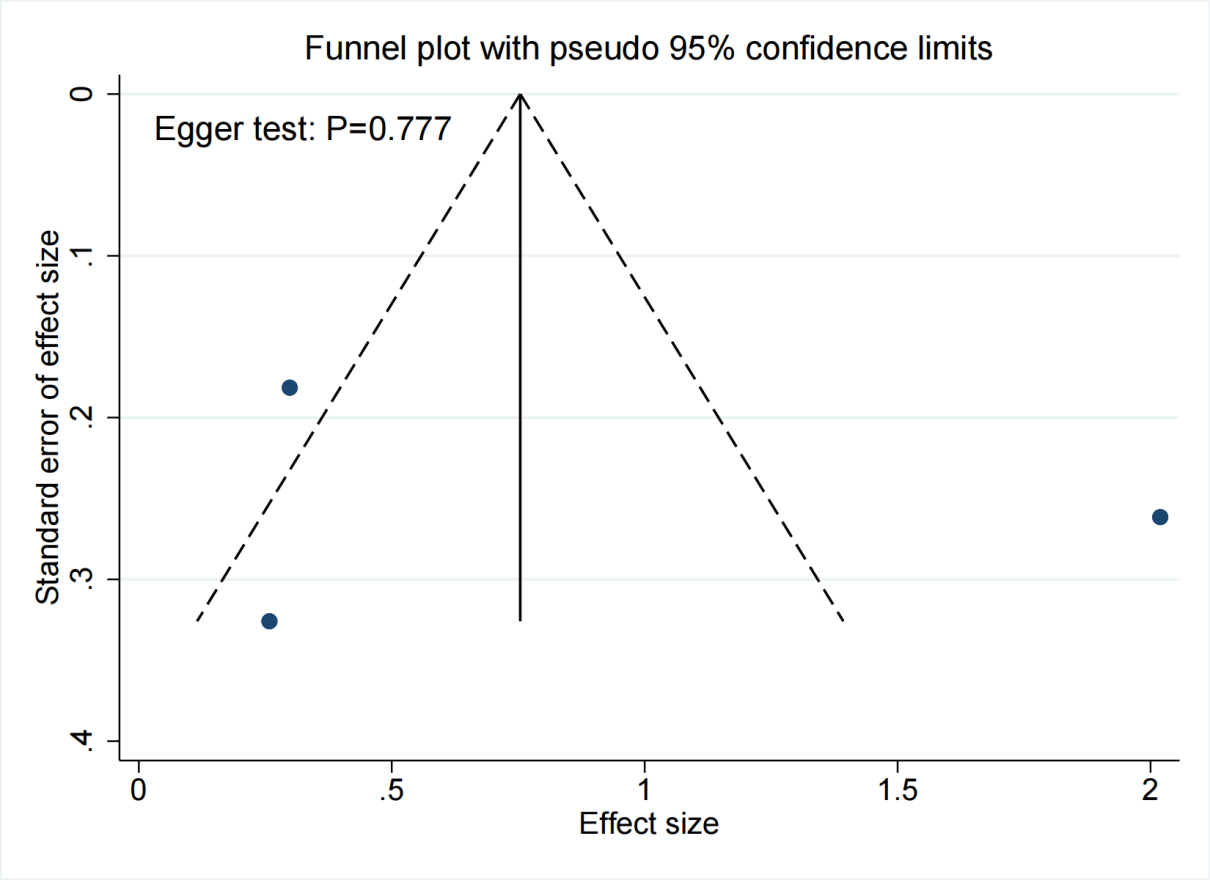


Supplementary figure 4.6 The funnel plot of sleep quality. The result of Egger test showed the p=0.777.
